# Characterization of patients with multiple sexually transmitted infections: A hospital-based survey

**DOI:** 10.4103/2589-0557.74978

**Published:** 2010

**Authors:** Shilpee Choudhry, V. G. Ramachandran, Shukla Das, S. N. Bhattacharya, Narendra Singh Mogha

**Affiliations:** Department of Microbiology, UCMS & GTB Hospital, Dilshad Garden, Delhi, India; 1 Department of Dermatology and STD, UCMS & GTB Hospital, Dilshad Garden, Delhi, India

**Keywords:** Commercial sex worker, multiple sexually transmitted infection, multivariate analysis, syndromic approach

## Abstract

**Background:**

Many studies have examined the inter-relationship between different STI. There are, however, a few data on patients presenting with more than one concurrent sexually transmitted infection (STI). The aim of the study was to determine the burden of patients with more than one concurrent STI and to characterize factors associated with such infections.

**Materials and Methods:**

Two hundred seventy five patients with one or more of the complaints, as enunciated by the World Health Organization (WHO) in its syndromic approach for the diagnosis of STI, were included as subjects. Detailed history, demographical data, and clinical features were recorded. All the patients were screened for common STIs. Multivariate analysis was performed taking all significant risk factor obtained from univariate analysis.

**Results:**

A total of 102 (37%) patients were identified as having multiple STIs amongst whom 72% (73/102) were male, 70% were married, and except one none reported regular use of condom The age of first sexual exposure was 13–17 years, 31.3% had more than three sexual partners in the past 6 months, and 76.4% had contact with commercial sex workers (CSWs). Multivariate analysis revealed statistical significance in relation to marital status, number of sexual partners exposed in the past 6 months, age of first sexual exposure, and age of patient at the time of presentation. Syphilis (48%) was the most common infection associated with multiple STI followed by HIV (45%) and HSV-2 (39.2%). None of the patients with multiple infections were detected and managed accurately by syndromic approach.

**Conclusion:**

Pattern of concurrent multiple STIs and the clinical severity of such multiple infections may serve as an indicator of the type of host–pathogen interaction determining the outcome of infection. When patient had multiple STIs, syndromic approach was not axiomatic and thus underscores the need for laboratory diagnosis.

## INTRODUCTION

It is a common practice in most sexually transmitted infection (STI) clinics that patients are offered screening for a variety of STIs, regardless of the initial presenting complaint. Many studies have been done to examine the inter-relationship between different STIs.[1] The association between STI and human immunodeficiency virus (HIV) has been well established.[2] Several studies have shown that the clinical manifestation of ulcerative STI facilitate the transmission of HIV.[3] A close link has also been demonstrated between chlamydial and gonococcal infection showing that the occurrence of chlamydia in individuals co-infected with gonorrhea is at a higher rate than would be normally expected.[1] There are, however, a few data on patients with more than one concurrent STI at the time of presentation.

The aim of this study was to determine the number of patients, with more than one concurrent STI, attending the STI clinic of a tertiary care hospital and to characterize factors associated with multiple infections. Such information might enable the clinician to recognize individuals who are at high risk of multiple infections and help health promoters to focus education more effectively on target groups. It may also enlist the extent of adequacy or otherwise of the pragmatic syndromic approach for instituting effective intervention.

## MATERIALS AND METHODS

Two hundred seventy five consecutive patients from April 2007 to March 2008 who attended STI clinic of a tertiary care hospital in Delhi, with one or more of the complaints as enunciated by WHO in its syndromic approach for the diagnosis of STI,[4] were included as subjects. Followed-up patients and asymptomatic patients were excluded from the study. Detailed history, demographical data, and clinical features were recorded from all the patients. All patients were managed on the basis of algorithms of the syndromic approach at peripheral health center (PHC) level recommended by the National AIDS Control Organization (NACO), India, after carrying out risk assessment.[5] Urethral and endocervical swabs were collected from males and females, respectively, and subjected to direct examination by Gram staining and culture plate inoculation at the site of sample collection. A presumptive diagnosis of gonococcal infection was made on observing polymorphonuclear leucocytes (PMNLs) with gram-negative intracellular diplococci (ICDC). If the smear showed five or more PMNLs in the absence of gram-negative ICDC, a presumptive diagnosis of non-gonococcal urethritis (NGU) was made in men.[6] For the isolation of *Neisseria gonorrhoeae*, swabs were directly inoculated on chocolate agar plate containing vancomycin, colistin, and amphotericin-B and incubated in 5–10% carbon dioxide for 24–48 h. Isolates were identified as *N. gonorrhoeae* on the basis of colony morphology, Gram staining, oxidase test, and rapid carbohydrate utilization test (RCUT).[7]

Normal saline wet mount examination was done to detect motile trophozoites of *Trichomonas vaginalis* and yeast cells for candida infection. For the isolation of Candida, urethral/cervical discharge was inoculated on Sabouraud dextrose agar and identification was done by standard mycological techniques.[8]

Direct smear was made from ulcer, if any, and subjected to direct examination by Gram staining and Leishman staining for the presence of multinucleated giant cells, shoals of fish bacilli, and safety-pin-appeared bacilli to detect herpes simplex virus (HSV), *Hemophilus ducreyi*, and *Calymmatobacterium granulomatis*, respectively.[8]

Ten milliliter of venous blood (without anticoagulant) was collected aseptically from all patients. Sera were separated and stored at −20°C in screw-capped glass tubes. HSV-2 IgM antibody in patient’s sera was detected by Ridascreen HSV-2 IgM (K5231, Germany) kit according to the manufacturer’s instructions. It is an indirect enzyme immunoassay for semi-quantitative estimation of IgM antibodies against the HSV type-2 in human serum. HSV-1 IgM antibody was also detected by Meddens Diagnostics HSV IgM μ capture EIA (REF 4051, Netherlands). It is an antibody class captured immunosorbent assay for the detection of HSV IgM in human serum. Sera were also tested for antibodies of other STIs namely Hepatitis B virus (HBV; 0003463 HEPALISA kit) and Hepatitis C virus (HCV; third generation HCV Microlisa kit, India) by ELISA and *Treponema pallidum* by venereal disease research laboratory (VDRL) test (antigen from the serologist of Kolkata, Government of India) followed by *T. pallidum* hemagglutination test (TPHA; Plasmatec TPHA test kit, Hansard diagnostic, UK). Antigen detection for *Chlamydia trachomatis* in genital swab of all the patients was performed by Bio-Rad Chlamydia Microplate EIA (31189 United States) kit. All patients were tested for HIV by ELISA/Rapid tests, using WHO approved kits following NACO guidelines, after pretest counseling and written informed consent, followed by post-test counseling. Genital wart and *Molluscum contagiosum* was detected clinically.

We tabulated and compared the differences in the risk factors among patients with two or more STI and the patients with single STI. For the qualitative variables we used Chi-square test. Unadjusted odds ratio (OR) and 95% confidence interval (CI) were calculated. Multivariate analysis was performed by using forward stepwise logistic regression, taking all significant risk factor obtained from the univariate analysis. Significance level was taken as *P* value <5%. For all analysis SAS Version 9 software was used.

## RESULTS

A total of 275 patients, with one or more STIs were included in the study. Of these, 102 (37%) patients were identified as having two or more STIs concurrently. Amongst patients having multiple infections, 72% (73/102) were male and male to female ratio was 2.5 : 1. Seventy percent of the patients were married and except one none reported regular use of condom Majority of patients (67.64%) were educated till the level of middle school while 26.47% were illiterate. The age at which patient had first sexual exposure ranged between 13 and 17 years. Also 31.3% patients (all males) had more than three sexual partners in the past 6 months and 76.4% had contact with commercial sex workers (CSWs). In our study group, there were 39 CSW amongst whom 18 had multiple STIs. One such patient was found to be reactive for 5 STI viz HBV, HCV, *T. pallidum*, HSV-2 and HPV.

Univariate analysis of the cases to assess the factors associated with acquiring multiple STIs revealed that there was significant difference between multiple STI and single STI groups with respect to the following parameters: age of patient, marital status, history of extra/pre-marital contact, number of partner exposed in the past 6 months, and age of first sexual exposure [Table 1]. These parameters were considered as plausible risk factors and were further subjected to multivariate analysis, and statistical significance was found in relation to marital status, number of sexual partners exposed in past 6 months, age of first sexual exposure, and age of patient at the time of presentation [Table 1].

**Table 1:** Significant factors associated with multiple STIs

			Univariate analysis		Multivariate	
Factors	Multiple STI (n = 102)	Single STI (n = 173)	OR (95% CI)	P-value	OR (95% CI)	*P* value
Sex			0.677 (0.398-1.149)	0.147		
Female	29	64				
Male	73	109				
Presenting complaint			0.773 (0.401-1.488)	0.605		
Others	24	44				
Discharge	42	78	0.763 (0.432-1.346)			
Ulcer	36	51	1			
Marital status			7.486 (4.320-12.971)	0.000*	11.89 (3.43-41.21)	0.000*
Married	72	42				
Unmarried	30	131				
Extra/pre-marital contact			4.403 (2.238-8.664)	0.000*		
Yes	90	109				
No	12	64				
No of partner exposed in past 6 month			9.429 (4.138-21.486)	0.000*	26.28 (4.03-171.10)	0.001*
						
≤3	70	165				
>3	32	8				
Nature of extra/pre-marital contact			1	0.000*		
No	12	64				
CSW	78	69	6. 028 3.004-12.097)			
						
Others	12	40	1.600 (0.655-3.905)			
Barrier contraceptive used			<0.122 (<0.016-0.946)	0.441		
No	101	160				
Yes	1	13				
Education			1.256 (0.421-3.742)	0.005*		
Illiterate	27	43				
1-12	69	112	1.314 (0.471-3.667)			
>12	6	18	1.000			
Age of patient	Mean = 29.09 ± 5.03	Mean = 27.36 ± 9.03		0.079	1.23 (1.12-1.34)	0.000*
Age of first sexual exposure	Mean = 15.65 ± 1.99	Mean = 20.15 ± 2.35		0.000*	0.14 (0.07-0.28)	0.000*

* significant *P*-value

The frequency of infection in the multiple STI group is shown in Table 2. Syphilis (48%) was the most common infection associated with multiple STI followed by HIV (45%) and HSV-2 (39.2%). Syphilis was predominantly associated with HIV (43%), HBV (40%), and genital wart (40%), while HSV-2 with HIV (46%). Patients having gonorrhea were found to be co-infected with HIV (20%), Chlamydia (60%), or/and HBV (17%) and had history of recurrent episodes of gonococcal infections. Patients presented with anogenital wart, concomitant infections were seen with HIV (66%), syphilis (66%), HSV-2 (16%), Hepatitis B (16%), and Hepatitis C (16%). In patients with HPV–HIV co-infection, the warts were multiple, diffuse and larger in size. None of the patients with multiple infections were detected and managed accurately by syndromic approach.

## DISCUSSION

Multiple STIs are common in patients who attend STI clinics. In our study, 37 percent of patients attending STI clinic had more than one concurrent STI. It is therefore important to screen individuals attending STI clinic for multiple STI irrespective of their presenting complaint.

In our study vast majority of the patients having multiple infections were male (72%) and had sub-optimal literacy, constituting the major bulk of patients with multiple STIs. The age at which patient had first sexual exposure ranged between 13 and 17 years. This age group represents the most vulnerable population for acquiring STIs. Women at this age are biologically more susceptible to acquire STI and the difference can be attributed to the epithelial lining of the cervix, as columnar epithelium in young women is more susceptible to infection and probability of developing neoplasia becomes more when exposed to human papillomavirus (HPV).[9]

The core group theory postulates that there is a central or ‘core’ group of individual who are infected with an STI and who can transmit the infection at a particular high rate thus maintaining the infection in a community by transmitting the infection to many partners.[10] Sex workers together with their clients form one such group. In our study group there were 39 CSW amongst whom 18 had multiple STIs. One such patient was found to be reactive for 5 STIs viz HBV, HCV, *T. pallidum*, HSV-2, and HPV. Also 76% of patients having multiple STIs had contact with CSWs, suggesting professional prostitution still remains the main source of STI among men having promiscuous behavior. Such study of STI prevalence in core groups are easier to monitor than trends in HIV seroprevalence and therefore valuable for determining the impact of HIV / AIDS control program. Epidemiological data so generated can identify targets for directing interventions and could be an effective means of controlling and reducing STI transmission in the population as a whole.

The multivariate analysis showed that age, marital status, number of partners exposed in the past 6 months, and age of first sexual exposure are significant factors associated with multiple STIs. Those who were married and had more than three sexual partners in past 6 months were more likely to have more than one STI. Also, younger the age of patient at the time of presentation and late his/her exposure to first sexual act, less is the likelihood of that individual acquiring multiple STI. Racial factors, socio-economic group, and sexual preferences were not taken into account as the vast majority of the patients, attending our clinics are Hindus, belonging to lower socio-economic group and heterosexual, thus their numbers would have been too small for statistical analysis.

Various studies have made observations about the inter-relationship between the different STI[1,10] but there is only a few data on burden and pattern of concomitant STIs. In the present study, it was observed that HSV-2 (39.2%), *T. pallidum* (48%), and HIV (45%) were most frequently associated with multiple STIs [Table 2]. This may be pertinent to their asymptomatic genital infection and secretion even after treatment.

**Table 2:** Frequency of different sexually transmitted infection in the multiple infections group

Infection	Frequency (n = 102)	Percentage
*T. pallidum*	49	48
HIV	46	45
HSV-2	40	39.2
HBV	26	25.4
HPV	20	19.6
*N. gonorrhoeae*	20	19.6
*C. trachomatis*	11	10.7
*T. vaginalis*	8	7.8
*C. albicans*	3	2.9
HCV/M. contagiosum	0	0

The presence of one STI may increase an individual’s chance of acquiring another STI. In our study, patients having syphilis had concomitant infection predominantly with HIV and genital wart. In the case of syphilis, infiltration of the dermis, underlying the primary chancre, with lymphocytes and plasma cells, may well facilitate the transmission of HIV.[11] Also the risk of contracting HIV infection is higher among persons with genital herpes and could result from a breakdown of physical barriers.[3] In our study also HSV-2 was predominantly associated with HIV (46%).

Gonococcal urethritis was found to be co-infected with HIV, Chlamydia and/or HBV infection. All these patients had recurrent episodes of gonococcal infections. Such recurrence of gonococcal infection in patients with multiple STIs highlights the vulnerability of such population in acquiring STI and also eventually becoming reservoir of drug-resistant organisms.

It is worth noting that in patients presenting with anogenital wart, other associated STIs were HIV, syphilis, HSV-2, Hepatitis B, and Hepatitis C. In patients with HPV–HIV co-infection, the warts were multiple, diffuse, and larger in size. HPV–HIV association resulted in quantitative and qualitative deviations in clinical disease that can be attributed to the host–pathogen interaction as mentioned above. Many recent studies have demonstrated a strong and consistent association between increased number of sex partners and increasing likelihood of anogenital wart.[12] Although these lesions most often spontaneously regress with no long-term side effects, risk factors especially in females that can lead to the development of cervical neoplasia include number of male sexual partner, age at first sexual intercourse, reproductive characteristics of the patients, smoking, and use of contraceptives.[13]

Although no concomitant infection was seen in patients having genital *Molluscum contagiosum*, it is a marker of co-existing STI and also HIV infection.[14] *M. contagiosum* thrives in the host by refusing to provoke the latter’s immune system, whereas HIV propagate better in an incited immune system. It might be interesting to see if such parasite association may subvert one another in a susceptible host.

Syndromic approach in large measure provides a pragmatic means of effective intervention. However, we observed that when patient had multiple STIs, this approach was not axiomatic, and treatment of these patients covered a single pathogen only, highlighting the inadequacy of the therapeutic coverage. Hence, while the practicality and field utility of syndromic approach is appreciated, at the same time, not to put too fine a point on it, this approach cannot totally extinguish the need for etiological diagnosis in the laboratory.

To conclude, pattern of co-infection and the clinical severity of such co-infection may serve as an indicator of the type of host–pathogen interaction determining the outcome of infection. In the present study, gonococci partnered with HIV, chlamydia and HBV, while HPV preferred to associate with HIV, HSV-2, and HCV besides *T. pallidum*. Such associations may not simply reflect an epidemiological association based on prevalent strain/agents in a particular setting but may be a pointer to an evolutionary principle of parasite interactions using the susceptible host as a milieu. Evidence for such notion may have to await long-term studies.
